# Improving antitumor efficacy via combinatorial regimens of oncolytic virotherapy

**DOI:** 10.1186/s12943-020-01275-6

**Published:** 2020-11-10

**Authors:** Bin Zhang, Ping Cheng

**Affiliations:** State Key Laboratory of Biotherapy and Cancer Center/Collaborative Innovation Center for Biotherapy, West China Hospital, Sichuan University, 17 People’s South Road, Chengdu, 610041 PR China

**Keywords:** Oncolytic virus, Oncolysis, Tumor tropism, Innate and adaptive immunity, Immunogenic cell death, Combination therapy, Antitumor

## Abstract

As a promising therapeutic strategy, oncolytic virotherapy has shown potent anticancer efficacy in numerous pre-clinical and clinical trials. Oncolytic viruses have the capacity for conditional-replication within carcinoma cells leading to cell death via multiple mechanisms, including direct lysis of neoplasms, induction of immunogenic cell death, and elicitation of innate and adaptive immunity. In addition, these viruses can be engineered to express cytokines or chemokines to alter tumor microenvironments. Combination of oncolytic virotherapy with other antitumor therapeutic modalities, such as chemotherapy and radiation therapy as well as cancer immunotherapy can be used to target a wider range of tumors and promote therapeutic efficacy. In this review, we outline the basic biological characteristics of oncolytic viruses and the underlying mechanisms that support their use as promising antitumor drugs. We also describe the enhanced efficacy attributed to virotherapy combined with other drugs for the treatment of cancer.

**Supplementary Information:**

The online version contains supplementary material available at 10.1186/s12943-020-01275-6.

## Introduction

Oncolytic virotherapy is an immunotherapeutic modality that utilizes naturally or genetically modified oncolytic viruses (OVs) to propagate in and selectively destroy carcinoma cells combined with a reduced capacity for infection and oncolysis of normal tissues and cells [1]. The unique characteristics of OVs in treating tumors have increased interest in oncolytic virotherapy research, with pre-clinical and clinical evaluation of a host of oncolytic virotherapies, including vesicular stomatitis virus (VSV) [2], adenovirus [3], vaccinia virus [4], and measles virus [5]. To date, only Talimogene laherparepvec (T-VEC), which is an attenuated herpes simplex virus type 1 (HSV-1) developed for the treatment of melanoma, has been approved by the Food and Drug Administration. In this oncolytic agent, the ICP34.5 and ICP47 regions have been deleted and granulocyte-macrophage colony-stimulating factor (GM-CSF) has been inserted [6].

For most viruses, a nucleic acid core composed of DNA or RNA and protein capsid (a nucleic coat) are integral to infection and proliferation, and, in some viruses, the lipid-rich envelope coating the capsid protein is required to promote viral attachment and entry into host cells. Oncolytic DNA viruses have high genome stability and large transgenes can be inserted into the viral vectors without impairing viral infection and replication function [7]. In contrast, most RNA viruses have limited genome packing capacity, and yet, are less likely to cause insertion mutations [8]. Therefore, various properties of viruses, such as the capacity to incorporate exogenous transgenes and copy stably, toxicity and immunogenicity, should be considered to optimize therapeutic efficiency of OVs.

Viruses have co-evolved with their hosts to develop sophisticated strategies for symbiosis and/or antagonization of the host immune system [9], which provides a favorable advantage for virus-based immunotherapy. The potent antitumor activity of OVs depends on not only their capacity for tumor tropism and direct oncolysis, but more importantly, their ability to engage the innate and adaptive immune responses [10]. However, given the potential antiviral machinery induced by activation of the interferon (IFN) signaling pathway [11] and the highly variable heterogeneity of malignant cells [12], OV-based monotherapy has restricted therapeutic effects. Perhaps not surprisingly, it is predicted that the superior therapeutic outcomes will be achieved through the combination of OVs with other standalone therapeutic strategies such as immunotherapy, chemotherapy or radiotherapy [7]. OVs can be genetically modified to encode transgenes of interest, thus virotherapy is a highly flexible platform, which offers benefits to versatile combination regimens. In this opinion article, we discuss the advantages and limitations of OVs, and explore how OVs preferentially replicate in tumors and affect host immune responses in multiple ways. Furthermore, we describe the marked benefits of OVs used in conjunction with other standard therapeutics, and explore how the combination provides mutual compensation for the shortcomings of each agent to have better efficacy.

## Multiple antitumor mechanisms of oncolytic virotherapy

During oncogenesis, tumor cells maintain uncontrollable cell reproduction by virtue of genetic and epigenetic changes that promote immune evasion, apoptosis inhibition and angiogenesis [12]. However, these growth benefits to the tumor come at the expense of the antiviral responses; hence tumors that are deficient in the machinery for viral clearance provide a permissive milieu for replication-competent viruses [13]. In addition to lysing cancerous cells, it has become clear that replication-selective OVs can stimulate systemic and durable antitumor immune responses by promoting the local release of antigens and cytokines [14]. Although potentiating antitumor immunity is generally considered to be the most effective mechanism of OV therapeutics, the relative contribution of each of the effects mediated by oncolytic virotherapy to the overall treatment outcomes remains uncertain. The therapeutic efficacy of OVs is likely to be correlated with a variety of mechanisms, such as the inherent properties of viral vectors and tumor cells, the activity of immune effector cells and the interplay between viruses, the tumor microenvironment and the patient’s immune system; however, these mechanisms remain to be fully elucidated [10]. Therefore, further investigations into the antitumor mechanisms underlying the effects of virotherapy are required to design optimal strategies for cancer treatment.

### Selective replication in tumor cells

Viruses have the ability to enter both normal and malignant cells. Although the antiviral machinery that exists in normal cells can detect and eliminate viruses, numerous cancerous cells lack this intrinsic machinery, providing an advantage for preferential replication of OVs within such cells. Certain viruses exhibit inherent tumor tropism; for example, reovirus replicate efficiently in tumors containing an abnormally activated RAS signaling pathway [15]. Activated RAS interferes with protein kinase R (PKR), a double-stranded RNA-activated protein, the phosphorylation of which inhibits protein translation, thus enabling synthesis of viral proteins [16]. The Edmonston strain of the measles virus has natural tropism for the human CD46 molecule that permits virus-cell binding and viral infection [17]. Despite the ubiquitous expression of CD46 in all nucleated cells, overexpression of CD46 in cancerous cells augments the susceptibility of tumors to the virus; hence, the measles virus exhibits oncolytic preference [18]. VSV blocks type I IFN production though the expression of matrix protein (M protein), which is reported to interfere with STAT activation [19], and/or host RNA and protein synthesis [20]. Therefore, VSV can replicate in IFN signaling pathway-deficient tumor cells [21].

Alternatively, conditional replication within neoplasms can also be accomplished by means of molecular modification of the viral genome using multiple approaches. A common approach facilitating exclusive replication of OVs in tumors is the generation of viral gene-deleted mutants in which gene regions toxic for normal tissues are deleted. For example, the γ34.5 gene of HSV-1 is a virulence gene, and the gene product counteracts PKR-mediated translation arrest by binding cellular protein phosphatase 1α (PP1α) and dephosphorylating eIF2α [22]. In general, the γ34.5 is deleted for brain tumors treatment because of its neurovirulence [23, 24]. In addition to attenuated neurovirulence, γ34.5 mutant oncolytic HSV-1 acquires replication competence in PKR-abnormal neoplastic cells, while normal cells are not permissive for viral replication due to translation shutoff and apoptosis following viral replication [25]. E1A-mutant oncolytic adenovirus is incapable of replication within normal cells because the intact cell cycle monitoring system interrupts the host protein synthesis on which viral survival depends; however, cancerous cells with defective cell cycle regulation are permissive to E1A mutant-induced S phase entry and synthesis of viral proteins, thus favoring viral tumor-selectivity [26]. Similarly, an oncolytic adenovirus with molecularly engineered deletion of EIB gene also exhibits tumor-selective replication in tumors with dysfunctional p53, but not in normal cells [27, 28].

Another approach enhancing viral tumor tropism involves insertion of specific genes targeting tumors and/or utilization of promoters that are exclusively activated in the tumor milieu to control selective expression of virulence genes. For example, it has been proposed that oncolytic HSV can be used to retarget tumors by replacing the natural receptor-binding regions of glycoprotein D with a single-chain variable fragment (scFv) specific for the human epidermal growth factor receptors (EGFRs) [29]. Moreover, a telomerase-specific oncolytic adenovirus exploits the human telomerase reverse transcriptase promoter to enable tumor selectivity of therapeutic genes [30, 31]. The oncolytic HSV-1 utilizes the nestin promoter to control the ICP34.5, leading to induction of tumor-specific viral propagation and oncolysis while retaining reduced virulence in normal cells [32]. Similarly, the major late promoter, survivin promoter and vascular endothelial growth factor (VEGF) promoter have also been shown to benefit selective replication of OVs in preclinical trials [33–35]. In addition, capsid modification can also enhance tumor targeting. For example, an adenovirus 5/3 chimera comprising the knob domain from serotype 3 facilitates virus entry into tumor cells, since tumor cells express high levels of adenovirus 3 receptors [36].

In summary, tumor-specific replication, which is integral to the role of OVs as novel antitumor agents, is dependent on multiple factors, including the inherent properties of cancer cells and OVs, the interaction between the two, and other factors present in the tumor microenvironment.

### Modulatory effects of oncolytic virus on immunological processes

#### Induction of immunogenic cell death

Tumor cells can undergo immunogenic cell death (ICD) under conditions of stress or damage. The key indicators of ICD include but are not limited to: release of ATP and nuclear high mobility group box 1 (HMBG1); cell surface exposure of calreticulin (CRT); and secretion of type I IFNs [37]. These marker molecules are referred to as danger-associated molecular patterns (DAMPs). As a result of ICD, dying cancer cells release pathogen-associated molecular patterns (PAMPs), DAMPs and tumor antigens, which attract inherent immune cells to the sites of lesions while activating immature dendritic cells (DCs), subsequently priming CD8^+^ T cells to produce a tumor-specific immune response.

Similar to some typical antitumor treatments, such as chemotherapy and radiation therapy, which are efficient ICD inducers [38, 39], OVs also have the capacity for ICD induction. For example, Newcastle disease virus (NDV) immunotherapy has been demonstrated to promote the translocation of CRT to the cell surface and extracellular accumulation of HMGB1 in orthotopic murine glioma models, along with tumor-specific immune response and durable tumor control [40]. Measles virus and coxsackievirus B3 can trigger release of analogous danger signal molecules that induce ICD of infected cells in vitro, which attracts abundant immune cells into the tumor microenvironment [41, 42]. In short, OVs induce immunogenic death of cancerous cells leading to the release of soluble antigens and inflammatory substances that promote activation of immune effector cells and priming of innate and adaptive antitumor immune responses.

#### Activation of innate immunity

A crucial step in the innate immune response is the initial detection of heterogeneous substances, a process that is reliant on pattern recognition receptors (PRRs), which are responsible for the surveillance of PAMPs and DAMPs [43]. PRRs and other related sensing factors include cyclic GMP-AMP synthase (cGAS)-stimulator of interferon genes (STING), retinoic acid-inducible gene I (RIG-I)-like receptors (RLRs), toll-like receptors (TLRs) and PKR. The failure of tumor regression in STING-knockout tumor-bearing mice suggests that this signaling pathway is correlated with immunity against tumors [44]. In fact, some viruses, such as inactivated vaccinia virus Ankara, can induce antitumor immunity via CD103^+^/CD8α^+^ DCs that depend on STING-mediated cytosolic DNA sensing [45]. In contrast to STING-dependent DCs  in the setting of oncolytic virotherapy, many cancerous cells exhibit aberrant cGAS-STING pathway signaling during oncogenesis. For example, STING signaling was found to be impaired in the majority of colorectal and ovarian cancer carcinoma cell lines examined in which the synthase cGAS was commonly silenced, which rendered these malignant cells more susceptible to OVs and favored viral replication and oncolysis [13, 46]. In the presence of appropriate signals, RLRs and TLRs respond to RNA and/or DNA ligands, driving the expression of IFNs and related genes via multiple cooperative immunomodulatory factors [43]. A previous study demonstrated that an antitumor measles virus vaccine allowed plasmacytoid DCs to produce cytokines via engagement of TLR-7 receptors [47]. Furthermore, TLR-3 signaling plays an essential role in the mechanism by which oncolytic reovirus alters inhibitory tumor microenvironment [48].

Generally, despite the risk of viruses being cleared, OVs tend to replicate in tumors by virtue of aberrant activation of oncogenic pathways and the immunodeficient tumor milieu, which can induce innate immune defense and enhance the resulting adaptive antitumor immunity.

#### Elicitation of adaptive immunity

Localized oncolytic virotherapy can create an inflammatory environment rich in tumor-associated antigens (TAAs), viral antigens, cytokines and chemokines, facilitating maturation of APCs. Studies have demonstrated that OVs can upregulate the expression of major histocompatibility complex (MHC) molecules on DCs as well as costimulatory/activation markers, such as CD40, CD80, and CD86 [49–51]. Although viral infection may initially elicit virus-specific immune responses, the cross-presentation pathway is activated in the presence of tumor antigens, which subsequently induces tumor-specific immune responses upon tumor antigen recognition. This antigen cross-presentation pathway is essential for activating CD8^+^ T cell responses against tumor antigens. For example, the generation of cytotoxic T cell response induced by vaccinia virus Ankara appears to be dependent on cross-priming of DCs, which obtain foreign antigens produced by other infected cells, instead of endogenous presentation and direct priming [52].

OVs can also promote T cell trafficking and enhance their infiltration into tumor beds via multiple mechanisms, including elicitation of type I IFN signaling and chemokines release in responses to viral antigens [53]. After reaching the site of tumor growth, immune effector cells kill tumor cells in response to antigen recognition, and moreover, tumor cells that were uninfected by OVs but express the same tumor antigens are also killed, which is characteristic of systemic immune responses [45]. In addition, the local secretion of perforins and granzymes by cytotoxic T cells also efficiently destroys neighboring malignant cells, even those that even lack antigen expression or exhibit mutated antigens [54].

Collectively, OVs are capable of reversing some carcinogenic effects and enhancing antigen processing and presentation, T cell activation, trafficking and killing, eventually yielding powerful immunotherapeutic efficacy.

## Challenges to successful oncolytic virotherapy

**Fig. 1:**
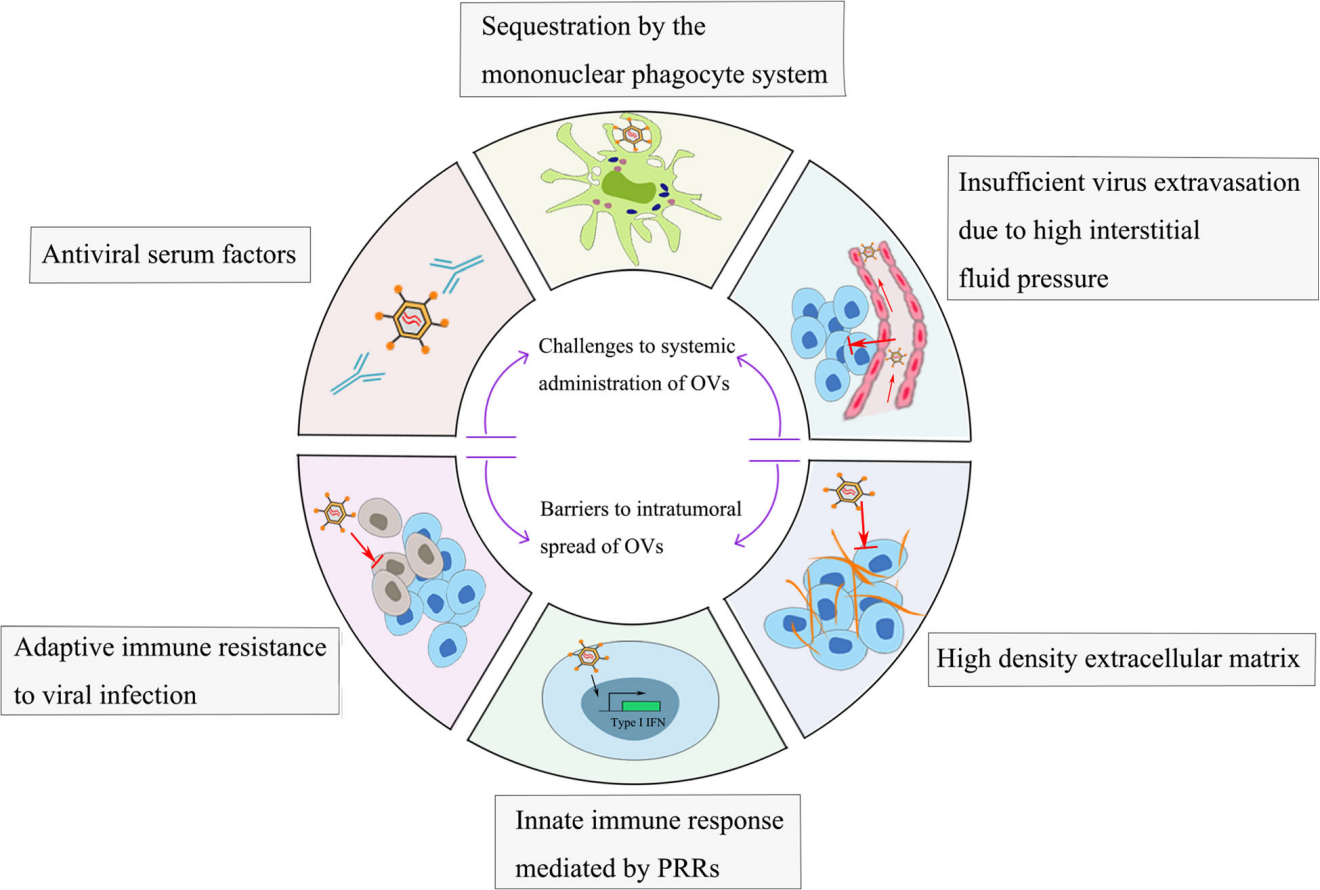
Limiting factors affecting the therapeutic effects of oncolytic virotherapy. Viral infection induces the generation of type 1 IFNs by PRR-mediated innate immunity. At the same time, tumor cells may shift sensitivity from the permissive status to a resistant status following durable virotherapy. The dense network of the extracellular matrix also hinders viral spread in tumors. Systemic delivery of naked therapeutic viruses may result in attenuation of viral activity and copies due to phagocytosis by the mononuclear phagocyte system and the neutralizing effects of serum antiviral factors. In addition, there is high interstitial fluid pressure in tumor tissues, which prevents effectively extravasation of the virus from the blood vessel

Despite the confirmed antitumor efficacy of oncolytic virotherapy, some challenges and obstacles facing OVs remain to be solved (Fig. 1). Limiting factors of OVs can be roughly classified into two aspects: 1) direct collapse of viruses and their life cycle through latent antiviral machinery, and 2) impeding viral functions indirectly by means of the intrinsic physical barriers and adaptive resistance of the surrounding milieu and tumors.

### Neutralizing antibodies and antiviral cytokines that attenuate virus activity

In the context of oncolytic virotherapy, the host immune system is the “frenemy” of OVs. One the one side, the therapeutic efficacy of OVs is dependent on potent antitumor immune responses, while in the other hand, antiviral immunity is a major obstacle to efficient oncolytic virotherapy. Pre-existing neutralizing antibodies and other antitumor serum factors can impair viral activity to some extent, and thus, it is difficult to have enough active viruses to reach the tumor site in the context of systemic delivery of naked OVs [55]. In addition, viral particles are detected by sensors on infected cells after viral infection, which in turn activate various type I IFN signaling pathways, such as DNA sensing cGAS-STING- or RNA sensing RLR- mediated signaling pathways. Type I IFN stimulates uninfected cells into a state of defense against the virus by inducing the expression of related genes; at the same time, it induces cell apoptosis and activates innate and adaptive immune cells to eliminate infected cells [56]. Rapid apoptosis or elimination of cancerous cells restrict viral spread, which is not conducive to the treatment of cancers with  OVs.

### Substantial barriers that hinder virus entry, infection and spread

Tumor cells have abnormal vascular structure manifested in high permeability and abnormal lymphatic networks, which leads to high interstitial fluid pressure in tumors [57]. This phenomenon may result in insufficient virus extravasation after intravenous administration of OVs. Moreover, interstitial hypertension is also linked to the abundant expression of extracellular matrix (ECM). The dense networks of ECM have also been demonstrated to be a substantial obstacle to prevent viral spread. For example, fibrillar collagen in the ECM limits oncolytic HSV spread within tumors, and matrix modulation by co-administration of OVs and bacterial collagenase improved the propagation of OVs [58]. Similarly, other ECM-degrading enzymes, such as hyaluronidase and metalloproteinases, have also been reported to enhance the distribution and potency of OVs [59, 60]. The blood-brain barrier, which prevents OVs reaching primary and metastatic brain tumors, is a cause of insufficient penetration of OVs into the central nervous system. In generally, most OVs are injected intratumorally in most preclinical and clinical studies; however, this administration route is limited to physically accessible tumors. Other modes of administration, such as intravenous and intraperitoneal delivery, are alternative approaches to intratumoral delivery [61, 62]; however, it should be noted that various administration modes need to be tailored the patients, disease and therapeutic viruses.

### Immune resistance to oncolytic virotherapy

Following years of research on cancer biology, it is now well established that tumor cells have evolved intricate machinery for immune evasion. The tumor environment contains an abundance of various types of immunosuppressive cells and inhibitory factors, such as tumor-associated macrophages (TAMs) and myeloid-derived suppressor cells (MDSCs), which secrete IL-10, transforming growth factor-β (TGFβ) and indoleamine-2,3-dioxygenase (IDO) to inhibit many important immunological processes [63, 64]. Therefore, it is crucial for OVs to maintain functions within the immunosuppressive tumor microenvironment. In addition, OV monotherapy can promote upregulation of the PD-1/PD-L1 axis on tumor cells and tumor-infiltrating immune cells [65, 66], which may dampen the therapeutic effect of oncolytic virotherapy. Indeed, oncolytic Maraba virus alone facilitated tumor-specific CD8^+^ T cell clonal expansion, although the magnitude of the immune response was insufficient because the T cell function was suppressed by the increased expression of PD-L1 [65]. Blockade of IFN signaling pathways markedly diminished PD-L1 expression on reovirus-infected glioma cells, indicating that OVs may induce PD-L1 in an IFN-dependent manner [67]. Furthermore, adaptive immune response have been shown to induce compensatory immunosuppressive pathways that augment the production of IDO and PD-L1, as well as attracting regulatory T cells (T_reg_) [68]. Of note, it is the immune system rather than cancer cells that drives these negative regulatory pathways. A recent study indicated that the inability of oncolytic NDV to induce tumor rejection is associated with this adaptive resistance. In this study, researchers observed that NDV alone enhanced effector T cell phenotypes but did not yield effectual tumor control, and further investigations revealed that NDV promoted PD-L1 production in the tumor milieu through distinct mechanisms, including augmented expression of PD-L1 occurred in virus-infected tumors as a response to virus-stimulated type I IFN signaling in a paracrine fashion, and in distant tumors as an adaptive immune resistance against increased tumor-infiltrating immune cells [69].

## Enhancing the antitumor effect by combination strategies including oncolytic virotherapy

**Fig. 2:**
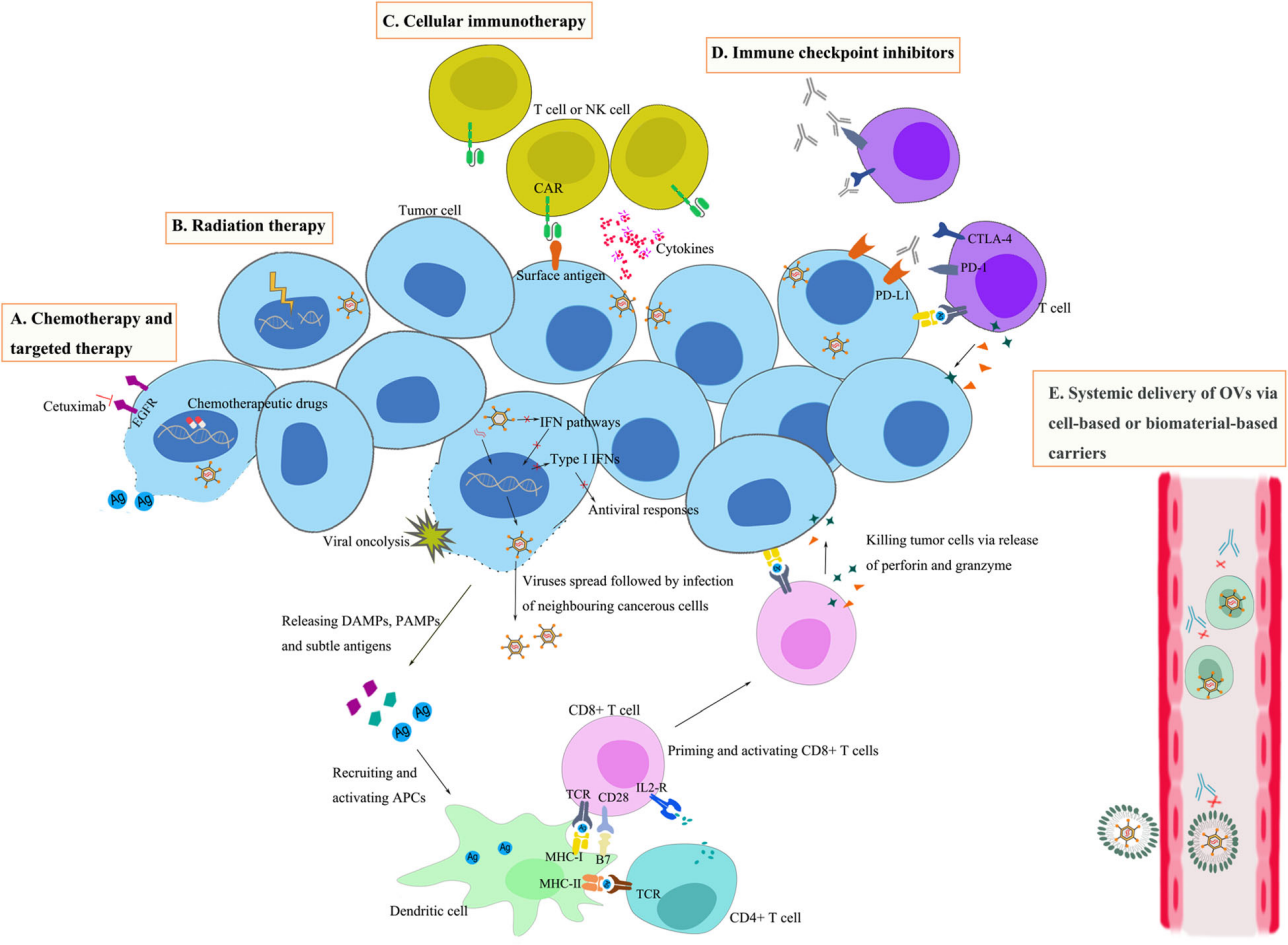
Oncolytic virotherapy as a combined platform of cancer treatment. OVs replicate selectively in tumor and have the capacity for direct oncolysis. More importantly, OVs induce immunogenic death of tumors followed by elicitation of immune responses, thus mediating a broader range of long-lasting antitumor effects. These characteristics of OVs provide a favorable platform for combination therapy in cancer. **a** Cytotoxic chemotherapy and molecular targeted therapy destroy tumors by termination of gene transcription and protein synthesis, or interruption of aberrant signaling pathways. Dying tumor cells release soluble antigens, resulting in increased expansion of the neoantigen repertoires induced by OVs and enhanced antitumor immunity. **b** Some OVs serve as radiation sensitizers by interruption of DNA damage repair and potentiating the sensitization of tumors to radionuclide therapy or external beam radiotherapy. Radiation therapy promotes enhancement of viral oncolysis. **c** OVs shape the tumor environment for immune cell therapy by shifting the tumor status from “cold” to “hot”, thus, improving immune cell recruitment and effector function. **d** Viral infection leads to increased expression of immune checkpoint molecules such as PD-L1 and CTLA-4, which augments the expression of therapeutic targets required for ICB and sensitizes infected tumors to ICB. In addition, OV-mediated increases in the release of DAMPs, PAMPs and cytokines promote the accumulation of cytotoxic T cells at tumor beds and retention of their killing capability. **e** Development of carrier systems, including cell- or biomaterial-based delivery systems, for transport of OVs is expected to reduce the impact of antiviral immunity on virus activity. At the same time, the ability of OVs to reach physically inaccessible tumors can be improved by systemic administration under the protection of carriers

OVs act on tumors directly or indirectly by means of multi-pronged antitumor mechanisms; hence, virotherapy provides an ideal therapeutic platform for cancer treatment (Fig. 2). Moreover, OVs represent an attractive combined platform by virtue of their engineering feasibility and confirmed safety profiles. Indeed, a host of combination strategies for natural or engineered OVs have been tested in the laboratory and clinical trials over recent decades (see Additional Table 1). In the section that follows, we describe the synergistic efficacy of OVs partnered with immunotherapy or other therapeutics. We highlight the rationale for combination strategies using OVs and how OVs overcome challenges associated with other antitumor treatments and potentiate overall therapeutic efficacy.

### Oncolytic virotherapy in combination with chemotherapy

Chemotherapy remains the current mainstream paradigms of cancer treatment. A combination of chemotherapy and oncolytic virotherapy enhances apoptosis induction, showing significant activity in a wide range of preclinical tumor models [70–72]. For example, gemcitabine partnered with an oncolytic adenovirus modified to express the extracellular matrix-degrading protein relaxin induced apoptosis in a pancreatic xenograft model, and also drastically attenuated the inhibitory effects of the matrix on viral spread and matrix-mediated resistance to chemotherapy, yielding effective tumor control [71]. In addition to their ability to enhance the induction of apoptosis, some chemotherapeutics (such as temozolomide) induce autophagy to potentiate oncolytic virotherapy by increasing virus replication [73]. Cancerous cells that are destroyed by treatment with cytotoxic chemotherapeutic drugs release DAMPs and soluble antigens. These effects can enhance the expansion of the neoantigen repertoires induced by OVs and promote antitumor immunity by inducting immunological death of cancer cells. Indeed, combination therapy with oncolytic HSV-1 plus mitoxantrone increased the accumulation of antigen-specific CD8^+^T cells within the tumor and improved therapeutic efficacy [74]. In parallel, a spectrum of clinical antitumor activity was demonstrated for this combination therapy [75, 76]. A phase I/II trial of carboplatin/paclitaxel plus reovirus showed synergistic cytotoxic activity and good objective responses in patients with head and neck carcinomas, accompanied by minimal antiviral immunity [75]. In another example, gemcitabine combined with an adenovirus expressing double-suicide genes was well tolerated and safe with evidence of activity in advanced pancreatic cancers [76].

### Oncolytic virotherapy in combination with radiation therapy

As one of the most common antitumor therapies, radiotherapy may cause radio-resistance or tumor recurrence, and damage to the surrounding normal tissues and cells. Utilizing the selective replication ability of OVs to promote the accumulation of radionuclides in tumor cells is conducive to increasing the precision and safety of the radiation treatment. Numerous studies have explored the broad-spectrum antitumor effects of radionuclide therapy in conjunction with oncolytic VSVs, HSVs, measles and other viruses that have been genetically modified to express the sodium iodide symporter (NIS), a membrane protein responsible for driving cellular uptake of radionuclides, such as ^131^I [77–80]. For example, administration of vaccinia virus expressing the NIS prior to ^131^I treatment increased the cellular concentration of radioiodine by intratumoral production of NIS proteins, and the dual treatment was more effective against prostate carcinoma cells compared to either OVs alone or ^131^I alone [81]. In addition to assisting radionuclide therapy to potentiate tumor targeting, OVs also play a significantly synergetic role in combination with external beam radiotherapy. An oncolytic NDV expressing an anti-CTLA4 antibody as a radio-enhancing agent synergized with standard radiation to boost tumor repression [82]. Ionizing radiation directly breaks DNA strands, leading to the production of DNA damage repair proteins, which are exploited for replication by some OVs. For instance, the ICP34.5 protein of HSV-1 is homologous to growth arrest and DNA damage protein 34 (GADD34), the expression of which is increased in response to radiotherapy in lung cancer; therefore, combined use of radiotherapy and oncolytic HSV-1 with the deletion of γ_1_34.5 promote virus replication and achieve synergistic efficacy [83]. At the same time, OVs are able to interrupt DNA damage repair and have potential adjuvant activity, thus, serving as radiation sensitizers. The adenovirus E4orf6 protein has been confirmed to inhibit the DNA repair mechanism and potentiate the susceptibility of solid tumors to external beam radiation [84]. Moreover, a triple combination therapy consisting of cisplatin plus radiation with intravenous delivery of oncolytic vaccinia virus was also found to be safe and feasible in a phase I trial conducted in patients with head/neck cancer [85].

### Oncolytic virotherapy in combination with molecular targeted therapy

Various small molecule compounds and biological antibodies have been designed to exclusively target abnormal signaling pathways and protein expression in tumors. Combined treatment with these targeted drugs and OVs presents a promising therapeutic strategy. Some agents targeting angiogenesis facilitate persistently high virus distribution throughout the tumor, enhancing the efficacy of oncolytic virotherapy. For example, combination therapy using the EGFR monoclonal antibody cetuximab, or the EGFR tyrosine kinase inhibitor erlotinib, with an oncolytic HSV-1 have been explored with generated synergistic tumor killing by enhancing the anti-angiogenic effect in human colorectal cancers and human pancreatic cancers respectively [86, 87]. Some refractory tumors may show reduced sensitivity to virus infection due to regulation by intrinsic signaling proteins, in this case, combination with targeted drugs can enhance cellular sensitivity. Malignant peripheral nerve sheath tumors (MPNSTs) were observed to resist infection of oncolytic HSV-1 through activation of the janus kinase (JAK)/signal transducer and activator of transcription 1 (STAT1) signaling pathway that drives constitutive expression of IFNs and resultant IFN-stimulated genes to diminish virus reproduction, as a result, co-treatment with the JAK inhibitor ruxolitinib improved the susceptibility of MPNSTs to oncolytic HSV-1 and showed superior antitumor activity over monotherapy [88, 89]. Similarly, inhibition of STAT3 allowed oncolytic VSVs to expand to high titers and reduced viral toxicity against primary hepatocytes, exerting synergy with VSV-based virotherapy for the treatment of hepatocellular carcinoma [90]. In addition, OVs in combination with molecular targeted agents promote the induction of cell apoptosis. Blockade of extracellular signal-regulated kinase (ERK) signaling with a BRAF or MEK inhibitor potentiated cell elimination in melanoma via cell apoptosis induced by ER stress when used in a therapeutic combination with a reovirus type 3 [91]. A γ34.5-deleted oncolytic HSV also exhibited synergistic effects with another anti-MEK molecular targeted drug [92]. Second mitochondrial activator of caspase (SMAC) mimetic compounds, which sensitize tumors to programmed cell death by thwarting the inhibitor of apoptosis (IAP) proteins, used in conjunction with an oncolytic rhabdovirus induced cytokine-mediated bystander cell death in vitro and provided additional efficacy in vivo [93]. Combination treatment with SMAC mimetics and oncolytic VSVs enhanced tumor regression via a CD8^+^ T cell-dependent mechanism [94]. Moreover, anticancer activity was heightened when OVs were used in combination with either rapamycin [95], a small molecule inhibitor of ataxia telangiectasia mutated protein (ATM) [96], or an agonistic antibody targeting immunostimulant 4-1BB [97].

### Oncolytic virotherapy in combination with immune checkpoint inhibitors

Immune checkpoint blockade (ICB) of checkpoint molecules such as programmed cell death protein 1 (PD-1) and its ligand (PD-L1) or cytotoxic T lymphocyte- associated protein 4 (CTLA-4) is used to reverse immune cell anergy by blocking immune-inhibitory signals. Despite the fact that ICB antibodies have been shown to offer a significant survival advantage for patients with various tumor types [98–100], some patients have low responses to the therapy [101]. This reality has led to focus of research on therapeutic strategies to improve ICB responses. OVs represent promising candidates that synergize with ICB to potentiate objective responses in patients with poor immunological infiltration.

#### Synergistic treatment with OVs and PD-1/PDL1 inhibitors

A straightforward factor leading to patients’ resistance to PD-1/PDL1 inhibitors is linked to the dearth of antigen recognition by T cells partly because of low mutation burden of tumor cells and/or defective antigen processing and presentation machinery. As mentioned previously, oncolytic virotherapy upregulates the PD-1/PD-L1 axis; hence, a combinatorial strategy consisting of OVs and PD-L1 blockade can augment the therapeutic targets required for the latter, while inhibiting PD-L1 can reduce potential immune resistance against oncolytic virotherapy, and generate more efficient antitumor activity [69]. Through analysis of the mutanome and immune status of lung adenocarcinoma cells, Norman and colleagues revealed that the tumor cells expressed multiple neoepitopes and a weak immune response was detected after PD-1 inhibition; however, injection of a modified oncolytic adenovirus elicited CD8^+^ T cell responses specific for neoantigens [66]. Moreover, intratumoral injection of OVs leads to immunological changes in the local tumor microenvironment with features of increased production of proinflammatory cytokines and chemokines as well as recruitment of immune effector cells, which increase the likelihood of refractory carcinomas response to PD-1/PD-L1 inhibitors and slow tumor growth under combination therapy [102–104]. A superior prognosis is closely correlated with the engagement of tumor-specific immune cells that could be elicited by the combination OVs and ICB. More importantly, this local antigen-specific antitumor activity can be extended to the whole body, that is, systemic antitumor immunity. For instance, A number of recent studies have shown that dual therapy of OVs and PD-1/PD-L1 inhibitors can promote amplification of cytotoxic effector T cells targeting a broad range of malignances, including malignant cells at both the injected and distant sites, consequently generating a potent systemic antitumor response [67, 105, 106]. Furthermore, following effective combined treatment, mice were protected against subsequent tumor cell challenge, with sustained long-term survival, which suggested the existence of efficiently acquired immune memory in the host that are important for durable defense against tumorigenesis [105, 107].

#### Synergistic treatment with OVs and CTLA-4 inhibitors

In addition to combination with PD-1/PD-L1 inhibitors, the combination of OVs and CTLA-4 blockade also provides an encouraging strategy. Many tumor types with poor immunological infiltration may not sensitive to CTLA-4 blockade at all; this is partly associated with the absence of tumor antigens as is the case with the challenges facing by simple PD-1/PD-L1 blockade. OVs induce CTLA-4 upregulation, making tumors sensitive to CTLA-4 blockade. For instance, Zamarin and colleagues found that CTLA-4 expression was upregulated after NDV infection, while analysis of the immunological characterization of tumor lesions revealed significant elevation of effector T cells to Treg ratios and increased frequencies of activated immune cells following combinatorial treatment of NDV and CTLA-4 inhibitors [108]. The OV-induced increase of T cell numbers within the tumor microenvironment can sensitive tumors to CTLA-4 blockade. Another study demonstrated the superior antitumor activity of the combination of anti-CTLA-4 antibody delivered systemically and oncolytic rotavirus administrated intratumorally. Even in a double-tumor mouse model of lymphoma or neuroblastoma, the combination achieved complete regression of both injected and abscopal tumors [107]. Several other strategies, such as treatment with an oncolytic vaccinia virus with deletion of the B18R gene deleted combined with CTLA-4 blockade, have also shown significant therapeutic responses in preclinical mouse tumor models [106].

#### Evidence for the therapeutic potential of the combined modality with OVs and ICB based on clinical data

The clinical efficacy of combined treatment has also been confirmed, for example, in a phase Ib clinical trial of T-VEC plus the anti-CTLA-4 antibody ipilimumab, with promising efficacy and tolerable safety profiles reported in patients with advanced melanoma [109]. In a subsequent phase II study, T-VEC coupled with ipilimumab also appeared to provide excellent therapeutic outcomes with a significantly higher objective response rate (38% vs. 18%) compared to that achieved by ipilimumab therapy alone, without increased toxicity or new adverse events [110]. In another example, in combination with systemic administration of anti-PD-1 antibody pembrolizumab, intratumoral injection of T-VEC altered the tumor environment and increased cytotoxic CD8^+^ T cell infiltration in melanoma patients. This combination therapy was characterized by elevated CD8α and IFN-γ mRNAs levels, and a good therapeutic effect in patients was confirmed on a phase Ib clinical trial, with overall and complete response rate was up to 62 and 33% separately [111].

Notably, in the case of certain combinatorial therapies, direct viral oncolysis may not be necessary for efficient tumor rejection. For example, an inactivated oncolytic rotavirus retained its synergistic effect with ICB irrespective of its deficiency in oncolytic activity [107]. Another study also demonstrated that tumors exhibit poor sensitivity to NDV-mediated cell lysis, but are highly susceptible to the combination therapy [108].

### Oncolytic virotherapy in combination with immune cell therapy

Apart from immune checkpoint inhibitors in the field of cancer immunotherapy, adoptive cell transfer therapy including natural immune cells and engineered immune cells, are also revolutionizing traditional cancer treatment modalities. Cellular immunology and oncolytic virotherapy can be combined to achieve better results by taking advantage of their complementary modes of action.

#### Synergistic effects of OVs and TIL therapy or TCR therapy

Tumor-infiltrating lymphocyte (TIL) therapy and engineered T cell receptor (TCR) therapy are based on their ability to recognize and eliminate cancerous cells that present their antigens in the context of MHCs. OVs have the ability to promote the expression of MHC molecules and other molecules involved in antigen processing [51], which is conducive to the synergistic effects of adoptive therapy with TILs or TCR-engineered T cells. TIL therapy is one form of cell therapy whereby naturally occurring T cells are harvested from patients’ tumors and then re-infused into patients following activation and expansion in vitro. The approach is currently being investigated with success in combination with oncolytic virotherapy in mouse models [112, 113]. Unfortunately, the lymphocytes isolated from some cancer patients may not be effectively expanded in sufficient numbers. In this case, engineered TCR therapy, whereby T cells are engineered to express a new T cell receptor that recognizes specific antigen targets, offers an alternative option to cancer treatment. For instance, the combination of TCR transgenic CD8+ T central memory cells with oncolytic VSV results in rapid tumor necrosis and exhibits a substantial therapeutic advantage compared with control T cells [114].

#### Synergistic effects of OVs and CAR-T cell therapy

In CAR-T therapy, T cells are equipped with chimeric antigen receptors (CARs), which have the ability to recognize and eliminate tumor cells even if tumor antigens are not presented in the context of MHCs. This therapy has a remarkable curative effect on hematologic malignancies. In particular, CD19-targeted CAR-T therapy has been extremely successful in treating patients with refractory B cell malignancies [115]. However, applying CAR-T cells for solid tumors is subject to challenges, and major limitations of the therapy include the immunosuppressive tumor microenvironment, which impedes CAR-T cell function through recruitment of immune suppressor cells and excessive expression of surface inhibitory molecules and also, the paucity of tumor-specific antigens essential for potent T cell responses [116].

It has been established that oncolytic virotherapy induces immunological infiltration in tumors, and thus, molecularly modified OVs that incorporate proinflammatory cytokines and/or chemokines can be co-opted to synergize with CAR-T therapy by reversing T cell anergy. For example, intratumoral delivery of the chemokine CXCL11 via a vaccinia virus vector led to accumulation of CAR-T cells in tumors and augmented the effect of CAR-T immunotherapy [117]. To enhance the migration ability and survival of CAR-T cells, inflammatory molecules secreted by the tumor mass, such as RANTES, can also be considered. Intratumoral injection of an oncolytic adenovirus armed with both RANTES and IL-15 enhanced the immune functions of GD2-specific CAR-T cells and contributed to prolonged survival of neuroblastoma-bearing mice [118]. Excessive expression of inhibitory immune checkpoint molecules on tumors hampers T cell function, which is another barrier to effective adoptive CAR-T cell therapy. Although antibodies targeting these suppressive molecules have a potent curative effect, they may cause systemic toxicity and side effects [119]. Therefore, local release of immunomodulators expressed from viral vectors is a safer and, perhaps, more efficacious method. An oncolytic adenovirus engineered to express a PD-L1 blocking antibody or a mini-body coupled with CAR-T therapy enhanced amplification and killing activity of HER2-specific CAR-T cells to yield potent solid tumor control [120, 121]. Antigen loss in solid tumors represents a third obstacle to CAR-T therapy and exploitation of bispecific T cell engagers (BiTE) represents a solution to this problem. Treatment with an adenovirus armed with an EGFR-targeting BiTE potentiated the proliferation and killing of folate receptor α (FRα)-specific CAR-T cells in vitro, moreover, the engineered adenovirus had the ability to direct FR.CAR-T cells to retarget EGFR in the absence of FRα on tumors [122]. As a result, the combinatorial therapy efficiently delayed tumor growth in a xenograft mouse model and had greater antitumor efficacy compared to the single agent therapy [122].

#### Synergistic effects of OVs and NK cell therapy

More recently, advances in cell therapy have enabled investigators to explore other immune cells, for example in NK cell immunotherapy. The dual therapy consisting of NK cells in conjunction with OVs is being explored in various types of tumor models. In the context of combination treatment, primary human NK cells activated by virus infected tumor cells augmented the killing and cytotoxicity of an oncolytic adenovirus in ovarian cancer [123], as well as an oncolytic measles virus in sarcoma cells [124]. NK cells can also be equipped with cancer-targeting CARs to specifically recognize tumor antigens. Studies have shown satisfactory efficacy of a combination of EGFR-targeting CAR-NK therapy and oncolytic HSV therapy [125]. Additionally, Yoo and colleagues reported that treatment of glioblastomas with proteasome inhibitor bortezomib before oncolytic HSV-1 infection prevented apoptotic cell death and instead induced inflammatory necroptosis [126]. Herein, the authors leveraged the proinflammatory features for NK cell immunotherapy, and as a result, combination therapy promoted NK cell activation and significantly enhanced NK cell adjuvant therapy [126]. A follow-up study investigated the effect of NK cells and used a mathematical model to predict the optimal density of NK cells in antitumor therapy combined with oncolytic HSV-1 and bortezomib [127].

### Arming oncolytic viruses with therapeutic genes

Oncolytic virotherapy is a flexible platform in which diverse transgenes of interest can be introduced into OVs by genetic modification. As a gene carrier, OVs can be used to safely deliver transgenes to tumor sites due to their tumor-selective replication, an advantage that helps avoid either uncontrolled off-tumor toxicity or other problems associated with systemic delivery.

#### Arming OVs with proinflammatory cytokines and chemokines

To date, multiple studies have investigated the use of OVs armed with proinflammatory cytokines and/or chemokines for in situ vaccination. A clear benefit of this arming strategy is that the immunomodulatory properties of cytokines provide benefits by “heating up” the tumor microenvironment, and the engagement of OV further elicits tumor-specific immune responses. Using this approach, typical cytokines, such as GM-CSF, promote DC recruitment and maturation and the therapeutic effects of delivery of GM-CSF by different viral backbones have been reported extensively [6, 128–130]. An oncolytic adenovirus coding for GM-CSF resulted in the induction of potent antitumor immunity and significant therapeutic effects in patients with solid tumors resistant to standard treatment [128]. Analogous examples include engineering OVs to encode interleukins, such as IL-12 or IL24, that function as inflammatory stimuli to promote immunological infiltration of the tumor microenvironment, which can strengthen efficacy when used in addition to other tumor therapeutics [131, 132].

#### Arming OVs with tumor antigens

Furthermore, arming OVs with a tumor antigen is an attractive strategy to elicit targeted immune responses of sufficient magnitude. In one example of this strategy, a recombinant vaccinia virus expressing human epidermal growth factor receptor 2 (HER2) elicited T cell responses with the release of IFN γ and IL-2 and induced rejection of a salivary gland tumor following vaccination [133]. Intratumoral co-treatment with separate vaccinia virus expressing GM-CSF and HER2 decreased levels of MDSCs and enhanced systemic antitumor activity in MDSC-rich tumors compared to treatment with either of the individual viruses alone [134]. It is also possible to incorporate a cytokine and TAA into the same viral vector [135]. Another notable approach involving TAA-encoding OVs is the heterologous prime-boost immune strategy that can focus immune responses toward the tumor antigens and away from viral antigens. This strategy has been demonstrated in several experimental models. For example, both potentiated prophylactic and therapeutic antimelanoma activity were observed as a result of priming with a VSV-based cancer vaccine encoding human dopachrome tautomerase (hDCT) followed by delivery of a booster dose with an adenovirus encoding the same antigen [136]. In accordance with this, immune responses primed by delivery of an adenovirus encoding hDCT were boosted rapidly following intravenous administration of a hDCT-expressing attenuated Maraba virus, with significantly extended survival in melanoma-bearing mice [137]. This approach was further advanced by combination therapy with immune checkpoint inhibitors and a prime-boost vaccination protocol using OVs [138].

#### Arming OVs with immune checkpoint inhibitors

In addition to cytokines and tumor antigens, delivery of checkpoint inhibitors by viral vectors provides another engineering strategy. Either a full-length antibody or scFv specific for PD-1/PD-L1 or CTLA-4 can be inserted into the viruses. This arming approach may theoretically produce therapeutic effects comparable to that of OV and ICB combinations, and importantly, the propensity for accumulation of OVs at the tumor site can circumvent the immune-related adverse events caused by systemic administration of ICBs. For example, in a preclinical human cancer xenograft tumor model, an oncolytic adenovirus expressing anti-CTLA-4 antibodies resulted in extremely high antibody concentrations at tumors, while plasma levels remain below concentrations reported tolerated in humans [139]. Furthermore, limiting the activity of those checkpoint inhibitors within the tumor can improve their therapeutic index and even obtain better antitumor activity. A study has demonstrated that a recombinant oncolytic myxoma virus expressing anti-PD-1 antibodies can not only exert an effectively effect in inhibiting tumor growth, but actually outperform the combination of PD-1 inhibitor and parental virus [140]. However, the local production of checkpoint inhibitor antibodies may not always be satisfactory, as the potential for ICB to maximize the therapeutic benefits requires the engagement of immune cells both in tumors and in the periphery. Therefore, combinatorial treatment using checkpoint inhibitor-expressing OVs and other anticancer agents is an appealing for optimizing cancer therapy [82, 120].

#### Arming OVs with a T cell engager

Bispecific T cell engagers, which consists of an anti-CD3 scFv fused with another scFv targeting a tumor cell surface antigen, are novel immunotherapeutic agents. BiTE-mediated tumor killing by T cells occurs in a TCR-independent fashion and without MHC presentation; however, the half-life of BiTE in serum is short, and there may be on-target off-tumor effect [141]. Leveraging OVs to deliver BiTE intratumorally under the control of a cell type-specific promoter provide an opportunity to avoid rapid BiTE metabolism and undesirable toxicities. Yu and colleagues first reported the therapeutic potential of BiTE-armed OVs in a preclinical trial of a combination of oncolytic vaccinia virus and a BiTE targeting tumor antigen EphA2 [142]. In this study, BiTE genes under the control of a late promoter did not impair oncolysis of the parental vaccinia virus, and furthermore, the BiTE-armed oncolytic vaccinia virus redirected T cells to EphA2-positive tumors while inducing bystander killing of adjacent tumors [142]. Similarly, an oncolytic adenovirus engineered to encode a BiTE specific for EGFR led to robust T cell activation, even in the absence of IL-2, compared with its parental counterpart [143]. Freedman and colleagues reported another promising result showing that a modified oncolytic adenovirus with EpCAM-targeting BiTE was capable of overcoming immune suppression and activating endogenous T cells [144].

### Systemic administration of oncolytic viruses in combination with delivery carriers

As mentioned previously, following systemic administration of naked viruses, the pre-existing neutralizing antibodies and virus-specific immunity in the host severely attenuate the copies and activity of most viruses prior to deposition at the tumor site. In this regard, development of carrier systems, such as cell- or biomaterial-based delivery systems for transport of OVs has attracted considerable attention.

#### Using cells as delivery vehicles for OVs

As a promising systemic delivery tool, carrier cells serve as “Trojan horses” that disguise therapeutic viruses from host immune defenses. As one of the best candidates for drug vehicles, stem cells including mesenchymal stem cells and neural stem cells, have been widely used due to several advantages, such as natural tumor homing properties and low immunogenicity [145–147]. Immune cells are also used as tools for the delivery of OVs. Previous reports have revealed that macrophages have the ability to migrate to hypoxic areas of tumors [148]; accordingly, some groups have taken advantage of the natural tumor accumulation for systemic delivery of macrophages co-transduced with adenovirus, leading to the localization of viruses in primary tumors and their metastases [149]. The same system used for delivery of adenovirus significantly prolonged survival in tumor-bearing mice following chemotherapy or irradiation therapy [150]. It has been proposed that leveraging a combination of T cells or DCs as delivery vehicles not only provided a protective effect against an oncolytic reovirus, but may also support the induction of innate and adaptive immunity [151, 152]. The virus-loading capacity and stability of carrier cells are crucial factors that determine the efficacy of oncolytic virotherapy. Several groups have examined chimeric oncolytic adenovirus with different fiber modifications, which were shown to enhance cellular internalization of viruses into carrier cells [146, 153]. Similarly, coating viruses with a biodegradable polymer enhances viral uptake into carrier cells [154]. Of note, the cytotoxicity of OVs on the carrier cells should be considered to prevent the cells being killed before they reached tumor sites [155].

#### Using biomaterials as delivery vehicles for OVs

Others have devoted similar efforts to develop biomaterial-based carrier systems as alternative systemic tools for the delivery of OVs. Avoiding virus neutralization is achievable by chemical or physical modification with various biomaterials. An example of this strategy is the encapsulation and coating of virion with liposomes [156, 157], nanovesicles [158], or polymers [159]. Some materials with stimuli-responsive properties may provide superior potential for systemic therapy, such as a pH-sensitive copolymer modified adenovirus for targeting the acidic tumor environment [160], and an enzyme-responsive liposome-coated adenovirus for reducing immunogenicity [161]. It has long been proposed that the enhanced permeability and retention (EPR) effect is responsible for non-specific transport of macromolecular drugs into solid tumors [162]; however, modification of biomaterials can also enhance the tumor tropism of carrier systems in a target-specific manner. For example, virus-liposome complexes carrying antibodies against CD71 and CD62E/P target activated vascular endothelium, and importantly, the addition of liposomes augments gene expression and cell internalization of viruses [163]. In another example, systemic administration of an adenovirus complexed with an EGFR-specific antibody-conjugated dendrimer targets EGFR-overexpressing tumors and shows potent therapeutic efficacy in an orthotopic lung tumor model [164].

## Conclusions

As highly promising cancer agents, OVs have shown significant benefits in the field of cancer treatment, which are primarily attributed to their unique capacity to induce oncolysis and immunomodulation. OVs engage the entire immunological process from detection to effect; however, despite multiple mechanisms, the dominant effects of OVs in determining overall efficacy remain unclear. Accumulating clinical data indicate that the potency of OVs is increased by combination with other anticancer drugs, especially cancer immunotherapy. OVs generally have good safety profiles because of their capacity for self-amplification in local tumors. Similarly, no added toxicity and adverse events were observed in clinical trials of combinatorial therapy with OVs and ICB compared with the effects of the individual agents [111]. Combining OV with other antitumor modality or arming it with an interest of gene, it should consider the relative merits of agents from the following aspects, including target of action, pharmacokinetic characteristics, safety profile, as well as cost of goods. With improved molecular understanding of the associated immunology, virology and tumor biology, it is expected more customized OVs and broad-spectrum combination strategies will be developed. Overall, oncolytic virotherapy is a promising and ideal therapeutic platform for optimizing combinatorial cancer treatments.

## Supplementary Information

**Additional file 1: Table 1.** Summary of current ongoing combination therapy trials of OVs on ClinicalTrial.gov.

## Data Availability

Not applicable.

## Abbreviations

OV: Oncolytic virus
VSV: Vesicular stomatitis virus
T-VEC: Talimogene laherparepvec
HSV: Herpes simplex virus
GM-CSF: Granulocyte-macrophage colony-stimulating factor
IFN: Interferon
PKR: Protein kinase R
scFv: Single-chain variable fragment
EGFRs: Human epidermal growth factor receptors
VEGF: Vascular endothelial growth factor
ICD: Immunogenic cell death
HMBG1: High mobility group box 1
CRT: Calreticulin
DAMPs: Danger-associated molecular patterns
PAMPs: Pathogen-associated molecular patterns
DCs: Dendritic cells
NDV: Newcastle disease virus
PRRs: Pattern recognition receptors
cGAS: Cyclic GMP-AMP synthase
STING: Stimulator of interferon genes
RIG-I: Retinoic acid-inducible gene I
RLRs: RIG-I-like receptors
TLRs: Toll-like receptors
TAAs: Tumor-associated antigens
MHC: Major histocompatibility complex
ECM: Extracellular matrix
TAMs: Tumor-associated macrophages
MDSCs: Myeloid-derived suppressor cells
TGFβ: Transforming growth factor-β
IDO: Indoleamine-2,3-dioxygenase
NIS: Sodium iodide symporter
MPNSTs: Malignant peripheral nerve sheath tumors
JAK: Janus kinase
STAT1: Signal transducer and activator of transcription 1
ERK: Extracellular signal-regulated kinase
SMAC: Second mitochondrial activator of caspase
IAP: Inhibitor of apoptosis
ATM: Ataxia telangiectasia mutated protein
ICB: Immune checkpoint blockade
PD-1: Programmed cell death protein 1
PD-L1: Programmed cell death protein ligand 1
CTLA-4: Cytotoxic T lymphocyte-associated protein 4
TIL: Tumor-infiltrating lymphocyte
TCR: T cell receptor
CARs: Chimeric antigen receptors
CAR-T: Chimeric antigen receptor-modified T cells
BiTE: Bispecific T cell engager
FR: Folate receptor
HER2: Human epidermal growth factor receptor 2
hDCT: Human dopachrome tautomerase
EPR effect: Enhanced permeability and retention effect
